# Ganodermanontriol Suppresses the Progression of Lung Adenocarcinoma by Activating CES2 to Enhance the Metabolism of Mycophenolate Mofetil

**DOI:** 10.4014/jmb.2306.06020

**Published:** 2023-10-01

**Authors:** Qingfeng Xie, Zhuo Cao, Weiling You, Xiaoping Cai, Mei Shen, Zhangyong Yin, Yiwei Jiang, Xin Wang, Siyu Ye

**Affiliations:** 1 Respiratory Department, Longquan People’s Hospital, No. 699, Dongcha Road, Longquan City, Zhejiang Province, 323000, P.R. China; 2 Respiratory Department, The Sixth Affiliated Hospital of Wenzhou Medical University, No. 15 Dazhong Street, Liandu District, Lishui City, Zhejiang Province, 323000, P.R. China; 3 Longquan People’s Hospital, No. 699, Dongcha Road, Longquan City, Zhejiang Province, 323000, P.R. China; 4 Wenzhou Medical University, Wenzhou Chashan Higher Education Park, Wenzhou, Zhejiang Province, 325006, P.R. China; 5 School of Public Administration, Wenzhou Medical University, Wenzhou Chashan Higher Education Park, Wenzhou, Zhejiang Province, 325006, P.R. China

**Keywords:** Lung adenocarcinoma, ganodermanontriol, mycophenolate mofetil, carboxylesterase 2

## Abstract

New anti-lung cancer therapies are urgently required to improve clinical outcomes. Since ganodermanontriol (GDNT) has been identified as a potential antineoplastic agent, its role in lung adenocarcinoma (LUAD) is investigated in this study. Concretely, lung cancer cells were treated with GDNT and/or mycophenolate mofetil (MMF), after which MTT assay, flow cytometry and Western blot were conducted. Following bioinformatics analysis, carboxylesterase 2 (CES2) was knocked down and rescue assays were carried out in vitro. Xenograft experiment was performed on mice, followed by drug administration, measurement of tumor growth and determination of CES2, IMPDH1 and IMPDH2 expressions. As a result, the viability of lung cancer cells was reduced by GDNT or MMF. GDNT enhanced the effects of MMF on suppressing viability, promoting apoptosis and inducing cell cycle arrest in lung cancer cells. GDNT up-regulated CES2 level, and strengthened the effects of MMF on down-regulating IMPDH1 and IMPDH2 levels in the cells. IMPDH1 and IMPDH2 were highly expressed in LUAD samples. CES2 was a potential target for GDNT. CES2 knockdown reversed the synergistic effect of GDNT and MMF against lung cancer in vitro. GDNT potentiated the role of MMF in inhibiting tumor growth and expressions of CES2 and IMPDH1/2 in lung cancer in vivo. Collectively, GDNT suppresses the progression of LUAD by activating CES2 to enhance the metabolism of MMF.

## Introduction

Lung cancer is the second most common malignancy in the world and the leading cause of death in cancer patients [1]. Clinically, lung adenocarcinoma (LUAD), as the most frequent pathological type of non-small cell lung cancer (NSCLC), accounts for 85% of all lung cancer cases, with main characteristics of dense lymphocytic infiltration and metastasis in the early stage [2, 3]. Despite breakthroughs in early diagnosis and standard treatments for lung cancer over the decades, most patients are already at an advanced stage when they are diagnosed [4]. Additionally, the overall prognosis of LUAD still remains poor, with 5-year survival rates ranging from 68% in patients at TNM stage Ib to 0-10% in patients at stages IVa-IVb, due to occurrence of relapse, drug resistance, side effects of chemotherapy, etc. [5, 6]. Therefore, it is urgently needed to develop novel antineoplastic agents with minimal side effects.

*Ganoderma lucidum* is a well-known precious Chinese traditional medicine, and possesses anti-inflammatory, antioxidant, hypoglycemic, anti-ulcer and anti-cancer properties [7, 8]. In recent years, a variety of active ingredients isolated from *G. lucidum* such as triterpenoids, polysaccharides and polypeptides have been confirmed to play important roles in both prevention and treatment of multiple cancers [9-11]. For example, *Ganoderma* triterpenoids in combination with gefitinib can attenuate tumor angiogenesis of lung cancer in vivo and concomitantly reduce gefitinib-caused side effects [12]. The study of Zhu *et al*. has highlighted the protective effect of *G. lucidum* polysaccharides on immune organs, and demonstrated that cisplatin combined with *G. lucidum* polysaccharides can alleviate cisplatin-induced immunosuppression and organ injury in cervical carcinoma tumor-bearing mice [13]. Thus, the development of adjuvant drugs based on *G. lucidum* ingredients is of great significance to enhance the anti-tumor efficacy and improve the prognosis of patients, and has attracted increasing interest. *Ganoderma*nontriol (GDNT) is one of the lanostane-type triterpenoids purified from *G. lucidum* [14]. Previous studies have demonstrated that GDNT can inhibit the malignant progression of breast cancer, colon cancer and hepatocellular carcinoma [15-17]. However, the potential role of GDNT in lung cancer still awaits investigation.

Carboxylesterase (CES) belongs to the serine hydrolase superfamily, and can hydrolyze endogenous compounds and exogenous chemicals in human body [18]. CES2 is predominantly expressed in the small intestines, and is instrumental in the metabolism of pharmaceutical products containing ester and amide bonds [19]. Growing evidence has supported that the genetic variant of CES2 is strongly associated with drug resistance and recurrence of some tumors [20, 21]. Mycophenolate mofetil (MMF) is an immunosuppressant widely used in clinical practice to treat acute rejection of transplanted organs [22, 23]. Recently, mycophenolic acid (MPA), the active metabolite of MMF, has been reported to restrict the growth and metastatic development of LUAD [24], but its molecular mechanism in regulating lung cancer is still elusive. There is evidence that MPA is an immunosuppressive drug targeting inosine-5’-monophosphate dehydrogenases (IMPDHs) [25]. IMPDHs can be increased in tumors, and down-regulation of IMPDH can serve as an anticancer therapy [26]. Despite the confirmed effect of IMPDH on small lung cancer cells [26], its role in LUAD has not been explored.

In the present study, we investigated the effect of GDNT or MMF on cell apoptosis and cycle, as well as on tumor growth in LUAD in vitro and in vivo. In addition, we further probed into the mechanism of GDNT in influencing the anti-cancer effect of MMF. Based on our findings, GDNT can enhance the inhibitory effect of MMF on the progression of LUAD by activating CES2 to increase the production of MPA.

## Methods

### Reagent Preparation

GDNT (E2500) was purchased from Selleck Chemicals (USA). MMF (HY-B0199) was ordered from MedChemExpress (USA). Dimethyl sulfoxide (DMSO, GC203005, Servicebio, China) was utilized to dissolve GDNT or MMF to prepare stock solution and stored at -20°C.

### Cell Culture and Treatment

Human lung cancer cell lines H1299 (CRL-5803) and A549 (CCL-185) were provided by the American Type Culture Collection (ATCC, USA). For cell culture, H1299 or A549 cells were grown in RPMI-1640 Medium (R8758, Sigma-Aldrich, USA) with supplement of 10% fetal bovine serum (10099141C, Thermo Fisher, USA) at 37°C in a humidified incubator (5% CO_2_, 95% air). For cell treatment, H1299 or A549 cells were incubated with GDNT at 0, 3.125, 6.25, 12.5, 25 or 50 μM, or with MMF at 0, 0.05, 0.5, 5, or 50 μg/ml in the culture medium for 24 h [15, 27].

### Bioinformatics Analysis

UALCAN database (http://ualcan.path.uab.edu/) was applied to analyze differential expressions of IMPDH1 or IMPDH2 in LUAD based on 59 normal samples and 515 primary tumor samples [28]. Swisstargetprediction database (http://www.swisstargetprediction.ch/) was employed to analyze putative targets for GDNT [29].

### Construction of Short Hairpin RNA (shRNA)

To knockdown CES2 in H1299 or A549 cells, shRNA targeting CES2 (shCES2; senses 5’-GGTCTCCAATTCTAGTTTA-3’, antisense: 5’-TAAACTAGAATTGGAGACC-3’) and its shRNA negative control (shNC) were synthesized by GenePharma (China). After culture, lung cancer cells were transferred into 6-well plates, and incubated overnight to reach 50-70% confluence. Then, cell transfection was performed with Escort IV Transfection Reagent (L3287, Sigma-Aldrich, USA) according to the manufacturer’s protocol. The cells transfected with shCES2 or shNC were harvested 48 h post transfection, and then subjected to examination of transfection efficiency using quantitative real-time reverse transcription polymerase chain reaction (qRT-PCR).

### RNA Isolation and qRT-PCR

Total RNA from lung cancer cells after cell transfection was isolated using RNeasy Mini Kit (74104, Qiagen, Germany). After quantification, complementary DNA (cDNA) was reversely transcribed from total RNA using RevertAid First Strand cDNA Synthesis Kit (K1622, Thermo Fisher, USA). The samples were mixed with Universal SYBR Green Supermix (1725120, Bio-Rad, USA), and then 7900HT Fast Real-Time PCR System (Applied Biosystems, USA) was used to conduct qRT-PCR. Primer sequences used in this reaction are as follows (5’-3’): CES2 (forward: CTAGGTCCGCTGCGATTTG, reverse: TGAGGTCCTGTAGACACATGG) and GAPDH (forward: GGAGCGAGATCCCTCCAAAAT, reverse: GGCTGTTGTCATACTTCTCATGG). The relative mRNA expression level of CES2 was calculated using 2^-ΔΔCt^ method [30], with GAPDH serving as the internal control.

### Methylthiazolyldiphenyl-Tetrazolium Bromide (MTT) Assay

Lung cancer cells with or without CES2 knockdown were seeded in 96-well plates (5 × 10^3^ cells/well) and incubated overnight. Next, the cells were treated with GDNT or MMF at different concentrations, or co-treated with 6.25 μM GDNT and 5 μg/ml MMF at 37°C for 24 h. Thereafter, 20 μl MTT solution (G4101-1, Servicebio, China) was added into each well for further 4-h incubation. Following formazan dissolution by DMSO, MD SpectraMax M5 microplate reader (Molecular Devices, USA) was utilized to detect cell absorbance at 570 nm.

### Assessment of Cell Apoptosis and Cycle

The determination of cell apoptosis was completed using Annexin V-FITC Apoptosis Detection Kit (CA1020, Solarbio, China). After cell transfection and/or cell treatment, lung cancer cells were suspended with 1×Binding Buffer at the concentration of 5 × 10^6^ cells/ml and later incubated with 5 μl Annexin V-FITC solution at room temperature (RT) for 5 min in the dark. Subsequently, cells were cultured with 5 μl propidium iodide (PI) and 400 μl phosphate buffered saline (PBS; G4202, Servicebio, China), and finally CytoFLEX SRT flow cytometer (Beckman Coulter, USA) was employed to analyze the apoptotic cells.

PI staining was carried out to evaluate cell cycle. According the manual of Cell Cycle and Apoptosis Analysis Kit (C1052, Beyotime, China), 2 × 10^5^ cells were suspended with pre-cooled PBS and fixed with 70% ethanol (G2350, Solarbio, China) at 4°C for 12 h. After 5 min of centrifugation (1,000 ×*g*), the cells were stained with prepared PI solution containing RNase A at 37°C bath for 30 min, followed by flow cytometry.

### Animals and Ethics Statement

Male immunodeficient nude mice (nu/nu, 6 weeks old) were reared in a controlled environment (26-28°C, 40-60% humidity, 12-h on/off light cycles) and allowed to access food and drinking water *ad libitum*. All animal experiments in this study were granted by Ethics Committee of Zhejiang Baiyue Biotech Co., Ltd for Experimental Animals Welfare (No. ZJBYLA-IACUC-20221026), and all procedures abided by the guidelines of the China Council on Animal Care and Use.

### Xenograft and Drug Administration

For performing in vivo xenograft, H1299 cells (5 × 10^6^) were suspended with 0.2 ml RPMI-1640 Medium, and injected subcutaneously into the right flank of each mouse. After 7 days of injection, mice were randomly divided into four groups (*n* = 4): Control group, MMF group (rats treated with MMF), GDNT group (rats treated with GDNT) and MMF + GDNT group (rats treated with MMF and GDNT). For drug administration, gavage of 20 mg/kg/2d MMF and/or intraperitoneal injection of 3 mg/kg/1d GNDT was carried out for 45 days, as described previously [16, 31]. Tumor volume was calculated every 9 days starting from day 18 based on the formula of (width × length)^2^ × 0.5. At last, animals were euthanized by cervical dislocation under deep anesthesia (5% isoflurane, 1349003, Sigma-Aldrich, USA). After being photographed, tumor samples were weighed and stored at -80°C until use.

### Immunohistochemistry (IHC)

Tumor samples were fixed with 10% formalin (E672001, Sangon Biotech, China) and embedded into paraffin according to standard procedures. Through microtomy, 5 μm sections were obtained, exposed to 3% hydrogen peroxide (H299581, Aladdin, China) for 20 min, washed with PBS and treated with 0.01 M citric acid (abs44109576, Absin, China). Subsequently, the sections were treated with 5% non-fat milk (abs9175, Absin, China) in 1×TBS with Tween-20 (TBST; G0004, Servicebio, China) at RT for 30 min. Then, the sections were incubated with CES2 antibody (PA5-102415, Thermo Fisher) at 4°C overnight, followed by hybridization with horseradish peroxidase (HRP)-conjugated mouse anti-rabbit IgG (D110065, Sangon Biotech, China) at RT for 1 h. After treatment with DAB (D12384, Sigma-Aldrich) and counterstaining with hematoxylin (G1004, Servicebio, China) in sequence, AXio Lab.A1 microscope (×100 magnification, Zeiss, ermany) was used to observe positive signals of CES2 in the sections.

### Western Blot

Cells or fresh tumor samples were homogenized in RIPA Buffer (R0010, China) as per the manufacturer’s specification to extract total protein which then was quantified by BCA Protein Assay Kit (PC0020, China). Equal amounts of protein samples (15 μg/lane) were separated using 10% SDS-PAGE and loaded onto polyvinylidene difluoride membranes (0.45 μm, 88585, Thermo Fisher), followed by blocking with TBST-diluted 5% non-fat milk (GC310001, Servicebio, China). Primary antibodies against CES2 (ab184957, 62 kDa), IMPDH1 (ab33039, 55 kDa), IMPDH2 (ab129165, 56 kDa) and GAPDH (ab181603, 36 kDa) were incubated with the membranes at 4°C overnight. Next, the membranes were hybridized with HRP-conjugated secondary antibody (ab6721) at RT for 1 h. All antibodies used in Western blot were provided by Abcam (UK). After that, the stripped membranes were treated with ECL Western Blotting Detection Kit (SW2040, Solarbio, China), and immunoblots were detected by ChemiDoc XRS+ imaging system (Bio-Rad). Bandscan software (Bio-Rad) was exploited to analyze relative protein expression (represented as the ratio of the gray-scale value of the target band to that of the GAPDH band).

### Statistical Analysis

Data from all experiments performed thrice were expressed as mean ± standard deviation. Comparisons between two groups or among multiple groups were carried out using independent-samples *t*-test or one-way analysis of variance, and the comparison of expression of the genes between normal and patient samples (Fig. 3A and 3B) was performed using non-paired sample *t* test. Statistical analysis was completed with Graphpad Prism 8.0 (GraphPad Software Inc., USA). Data with *p* value less than 0.05 were defined as statistically significant.

## Results

### GDNT Enhanced the Effects of MMF on Suppressing Viability, Promoting Apoptosis and Inducing Cell Cycle Arrest in Lung Cancer Cells

**Fig. 1:**
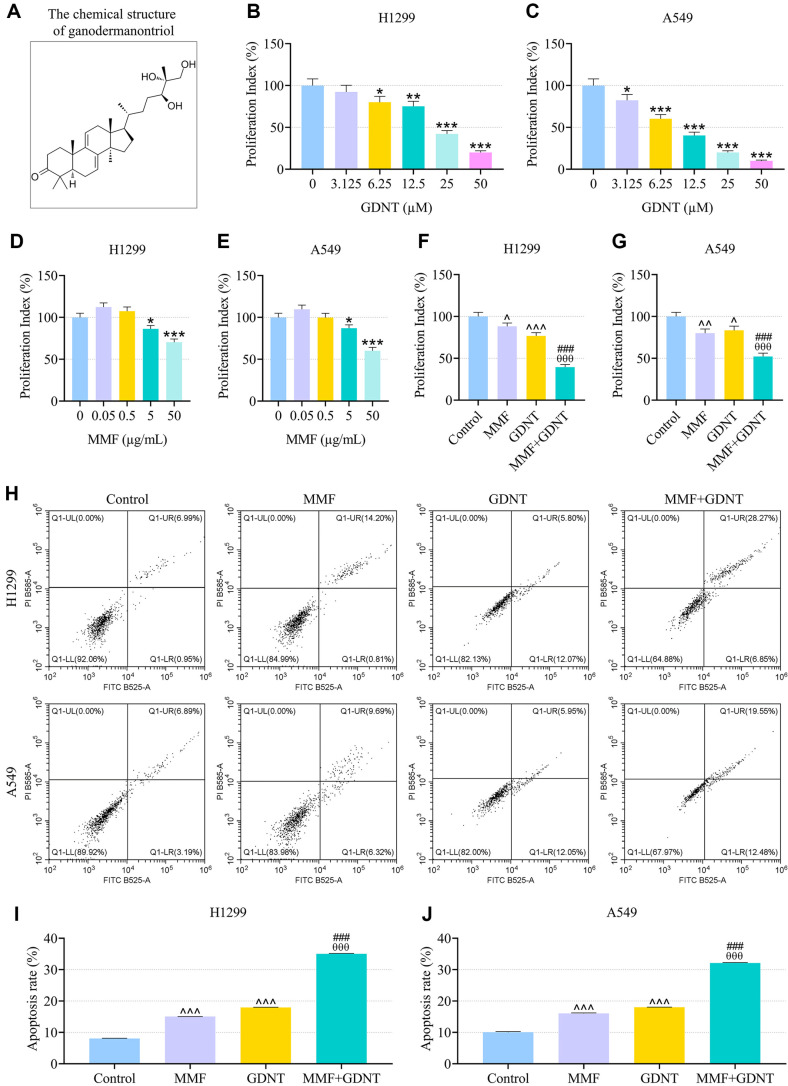
The effects of GDNT and/or MMF on viability and apoptosis of lung cancer cells. (**A**) The chemical structure of GDNT. (**B-E**) H1299 or A549 cells were treated with GDNT (0, 3.125, 6.25, 12.5, 25, 50 μM) or MMF (0, 0.05, 0.5, 5, 50 μg/ml) for 24 h, and MTT assay was performed to examine cell viability. (**F, G**) H1299 or A549 cells were treated with 6.25 μM GDNT and/or 5 μg/ml MMF for 24 h, and MTT assay was conducted to test cell viability. (**H-J**) After treatment with 6.25 μM GDNT and/or 5 μg/ml MMF for 24 h, flow cytometry was used to analyze cell apoptosis. The horizontal axis in the H graph represented the number of FITC stained cells, while the vertical axis represented the number of PI stained cells. Data from all experiments performed thrice were expressed as mean ± standard deviation. *p < 0.05, **p < 0.01, ***p < 0.001, vs. 0; ^*p* < 0.05, ^^*p* < 0.01, ^^^*p* < 0.001, vs. Control; ^θθθ^*p* < 0.001, vs. MMF; ^###^*p* < 0.001, vs. GDNT. MMF, mycophenolate mofetil; GDNT, ganodermanontriol; MTT, methylthiazolyldiphenyl-tetrazolium bromide.

**Fig. 2:**
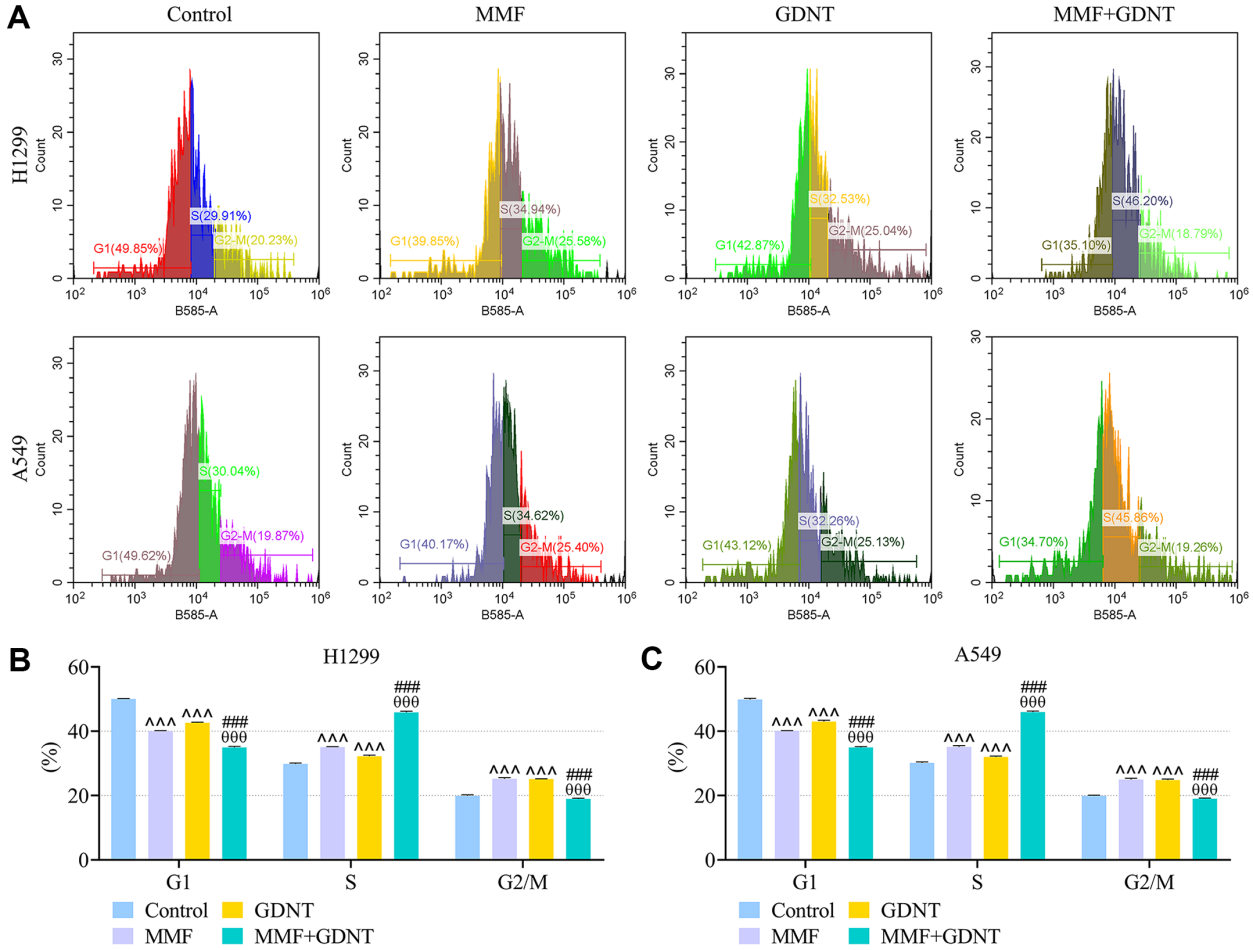
The effect of GDNT and/or MMF on lung cancer cell cycle. (**A**) H1299 or A549 cells were treated with 6.25 μM GDNT and/or 5 μg/ml MMF for 24 h, and flow cytometry was applied to analyze cell cycle. (**B, C**) Bar chart of cell cycle proportion in the Control, GDNT, MMF and MMF+GDNT groups. Data from all experiments performed thrice were expressed as mean ± standard deviation. ^^^*p* < 0.001, vs. Control; ^θθθ^*p* < 0.001, vs. MMF; ^###^*p* < 0.001, vs. GDNT.

Previous evidence has shown that GDNT (Fig. 1A), a lanostanoid triterpene extracted from *G. lucidum*, exerts antineoplastic bioactivity against some cancers [32]. To evaluate the potential effect of GDNT on lung cancer, H1299 and A549 cells were treated with different concentrations of GDNT for 24 h. As described in Fig. 1B-1C, the viability of H1299 cells was significantly decreased by GDNT at 6.25, 12.5, 25 and 50 μM (*p* < 0.05), and the viability of A549 cells was notably decreased by GDNT at 3.125, 6.25, 12.5, 25 and 50 μM (*p* < 0.05). Despite evidence supporting the ability of MPA (the active metabolite of MMF) to prevent the malignant progression of LUAD [24], the role of MMF in lung cancer still remains unclear. After 24-h treatment with different concentrations of MMF, the viability of H1299 and A549 cells was markedly decreased by MMF at 5 and 50 μg/ml (Fig. 1D-1E, *p* < 0.05). Based on the inhibitory effect of drug at different concentrations on cell activity as described above, 6.25 μM GDNT and 5 μg/ml MMF were used for subsequent in vitro experiments. Compared with the cells treated with GDNT or MMF alone, cells co-treated with GDNT and MMF exhibited impaired viability (Fig. 1F-1G, *p* < 0.001). According to flow cytometry analysis results, compared to control cells, either MMF or GDNT increased the apoptosis rate of lung cancer cells (Fig. 1I-1J, *p* < 0.001), reduced cell-cycle distribution in G1 phase and induced cell cycle arrest in S and G2/M phases (Fig. 2A-2C, *p* < 0.001). Relative to treatment with GDNT or MMF alone, co-treatment with GDNT and MMF further accelerated cell apoptosis and promoted cell cycle arrest in S phase (Fig. 2A-2C, *p* < 0.001).

### IMPDH1 and IMPDH2 Were Highly Expressed in LUAD Samples and CES2 Was a Potential Target for GDNT

**Fig. 3:**
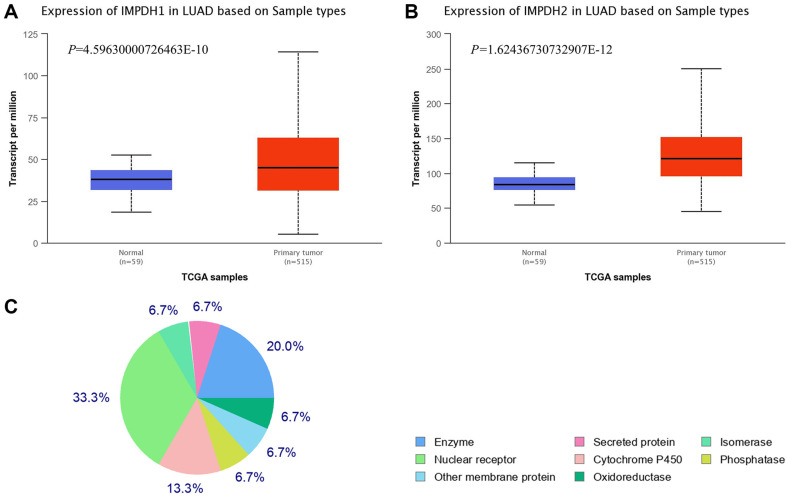
The expression of IMPDH1/2 in LUAD and the target prediction of GDNT. (**A, B**) Expressions of IMPDH1 and IMPDH2 in LUAD based on 59 normal samples and 515 primary tumor samples were analyzed using UALCAN database (http://ualcan.path.uab.edu/). (**C**) Swisstargetprediction database (http://www.swisstargetprediction.ch/) was employed to analyze putative targets for GDNT.

Based on TCGA, UALCAN database predicted that IMPDH1 expression was higher in primary tumors from LUAD samples than in normal samples (Fig. 3A, *p* = 4.59630000726463E-10). The predicted expression pattern of IMPDH2 in LUAD samples was similar to that of IMPDH1 (Fig. 3B, *p* = 1.62436730732907E-12). Later, we analyzed target genes of GDNT using Swisstargetprediction database, in which nuclear receptor (33.3%), enzyme (20%), and cytochrome P450 (13.3%) account for the largest proportion, and other targets share the same proportion (6.7%). According to the predicted results (Fig. 3C), CES2 (an enzyme) with the highest probability (Fig. S1) was considered as a potential target for GDNT as it has the highest score.

### GDNT Up-Regulated CES2 Expression and Enhanced the Effect of MMF on Down-Regulating IMPDH1 and IMPDH2 Expressions in Lung Cancer Cells

**Fig. 4:**
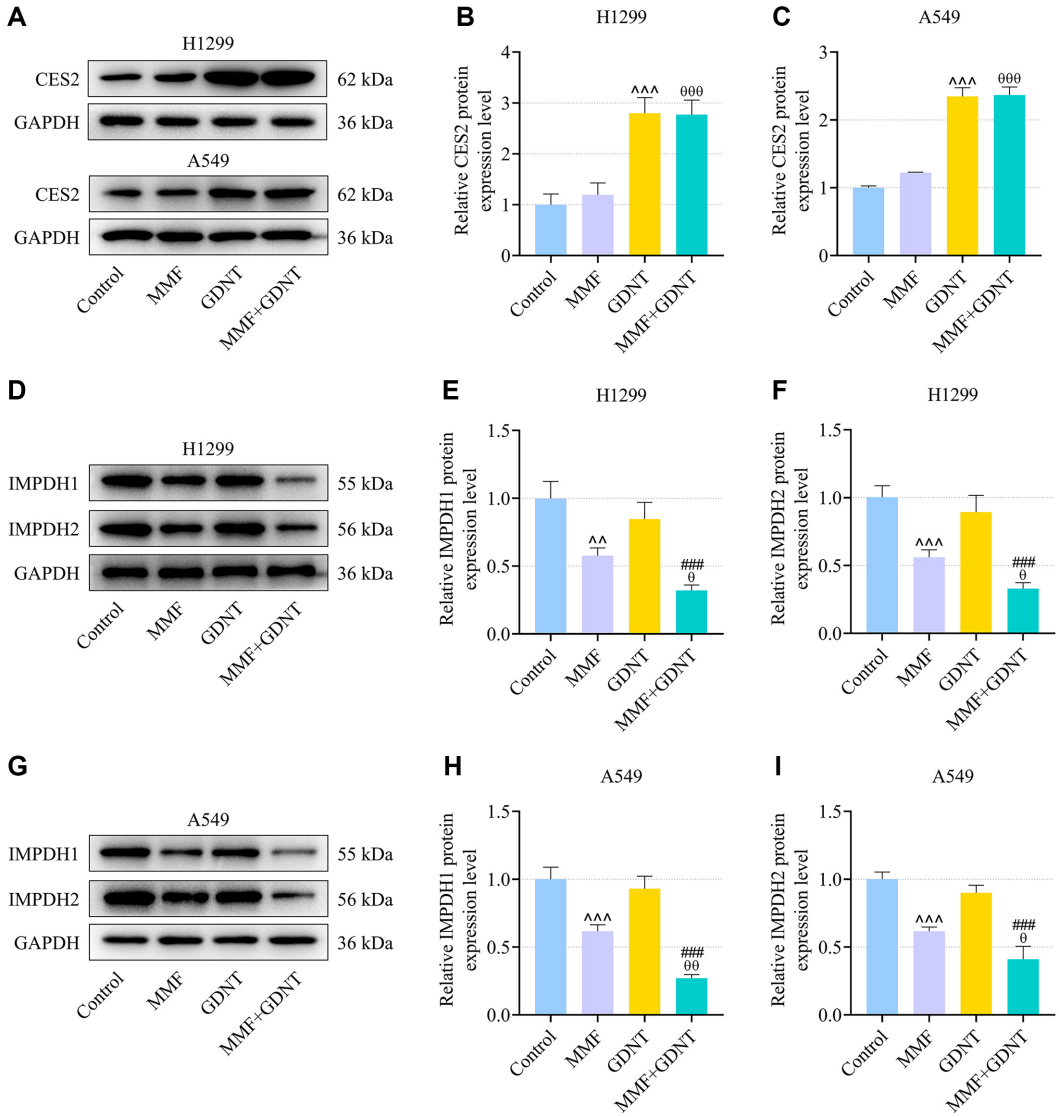
The effect of GDNT and/or MMF on CES2, IMPDH1 and IMPDH2 expressions in lung cancer cells. (**A-C**) H1299 or A549 cells were treated with 6.25 μM GDNT and/or 5 μg/ml MMF for 24 h, and Western blot was performed to detect protein level of CES2 in the Control, GDNT, MMF and MMF+GDNT groups. (**D-F**) After the cell treatment for 24 h, the protein expression levels of IMPDH1 and IMPDH2 in H1299 cells were measured by Western blot. (**G-I**) Following the cell treatment for 24 h, IMPDH1 and IMPDH2 protein expressions in A549 cells were determined by Western blot. Relative expression levels were normalized with GAPDH. Data from all experiments performed thrice were expressed as mean ± standard deviation. ^^*p* < 0.01, ^^^*p* < 0.001, vs. Control; ^θ^*p* < 0.05, ^θθ^*p* < 0.01, ^θθθ^*p* < 0.001, vs. MMF; ^###^*p* < 0.001, vs. GDNT. CES2, carboxylesterase 2; IMPDH, inosine-5’-monophosphate dehydrogenase.

CES2 has been indicated to influence MMF metabolism, given the genetic polymorphism of this metabolic enzyme [22]. In H1299 or A549 cells, it was determined that the protein level of CES2 was up-regulated by GDNT treatment (Fig. 4A-4C, *p* < 0.001). CES2 protein level was barely affected by MMF treatment, but was increased by co-treatment of MMF and GDNT (Fig. 4A-4C, *p* < 0.001). As demonstrated in Fig. 4D-4I, MMF down-regulated IMPDH1 and IMPDH2 protein levels in the cells (*p* < 0.01), the effect of which was potentiated by GDNT (*p* < 0.05). GDNT treatment alone had no significant effect on IMPDH1 and IMPDH2 protein levels in the cells, which, however, in combination with MMF can decrease the protein levels of IMPDH1 and IMPDH2 (Fig. 4D-4I, *p* < 0.001).

### CES2 Knockdown Reversed the Synergistic Effect of GDNT and MMF against Lung Cancer In Vitro

**Fig. 5:**
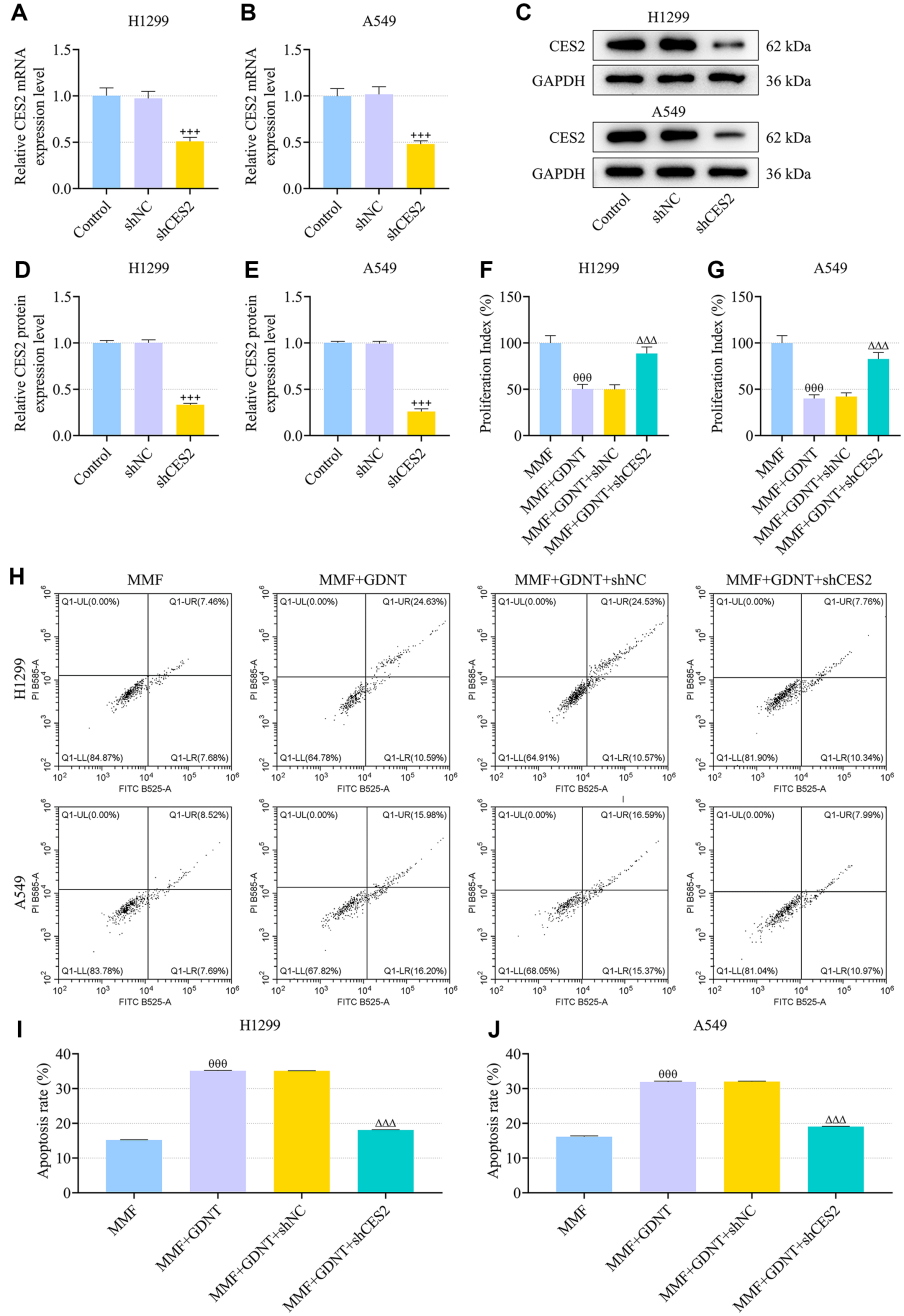
CES2 knockdown reversed the synergistic effect of GDNT and MMF on viability and apoptosis of lung cancer cells. (**A, B**) H1299 or A549 cells were transfected with shCES2 or shNC for 48 h, and qRT-PCR was performed to examine the mRNA expression of CES2, with GAPDH used as the internal control. (**C-E**) The protein expression of CES2 in the Control, shNC and shCES2 groups was determined by Western blot, with GAPDH used as the internal control. (**F, G**) After cell transfection and co-treatment with 6.25 μM GDNT and/or 5 μg/ml MMF, the viability of H1299 and A549 cells was evaluated by MTT assay. (**H-J**) The apoptosis of the indicated cells was tested by flow cytometry. Data from all experiments performed thrice were expressed as mean ± standard deviation. ^+++^*p* < 0.001, vs. shNC; ^θθθ^*p* < 0.001, vs. MMF; ^ΔΔΔ^*p* < 0.001, vs. MMF + GDNT + shNC. shCES2, short hairpin RNA (shRNA) targeting CES2; shNC, shRNA negative control.

**Fig. 6:**
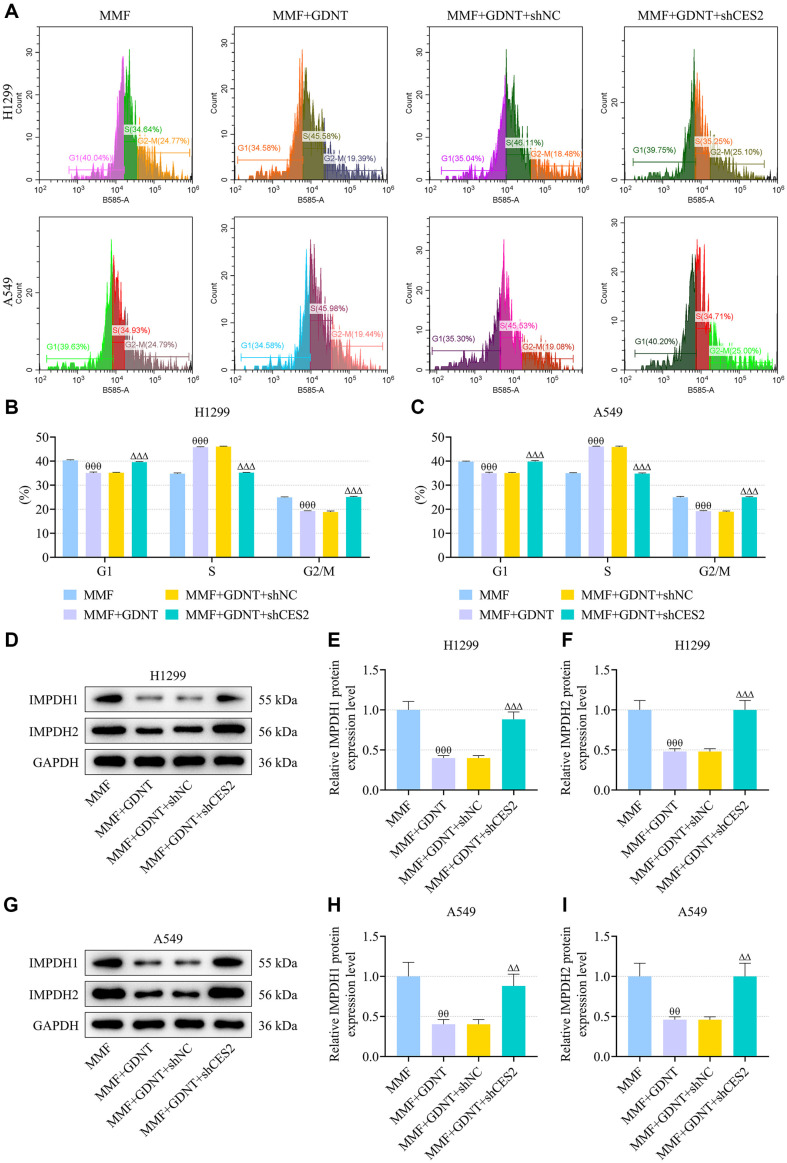
CES2 knockdown offset the synergistic effect of GDNT and MMF on cell cycle and IMPDH1/2 expression in lung cancer cells. (**A-C**) After cell transfection with shCES2 and co-treatment with 6.25 μM GDNT and/or 5 μg/ml MMF, the cell cycle of H1299 and A549 cells was analyzed by flow cytometry. (**D-I**) Protein levels of IMPDH1 and IMPDH2 in the indicated cells were measured by Western blot. Relative expression levels were normalized with GAPDH. Data from all experiments performed thrice were expressed as mean ± standard deviation. ^θθ^*p* < 0.01, ^θθθ^*p* < 0.001, vs. MMF; ^ΔΔ^*p* < 0.01, ^ΔΔΔ^*p* < 0.001, vs. MMF + GDNT + shNC. shCES2, short hairpin RNA (shRNA) targeting CES2; shNC, shRNA negative control.

Next, we explored the effect of CES2 on the development of lung cancer cells in the presence of MMF and GDNT. After transfection with shCES2, the mRNA and protein expressions of CES2 in either H1299 or A549 cells were strikingly decreased (Fig. 5A-5E, *p* < 0.001), as compared with the effect of transfection with shNC. Of note, results of following cellular functional assays confirmed that CES2 knockdown enhanced cell proliferation, repressed cell apoptosis and suppressed cell cycle arrest in S phase, which reversed the synergistic effect of GDNT and MMF on lung cancer cells (Fig. 5F-6C, *p* < 0.001). In cells co-treated with MMF and GDNT, CES2 silencing up-regulated IMPDH1 and IMPDH2 protein levels, as determined by Western blot (Fig. 6D-6I, *p* < 0.01).

### GDNT Potentiated the Tumor-Suppressive Effect of MMF on Lung Cancer In Vivo

**Fig. 7:**
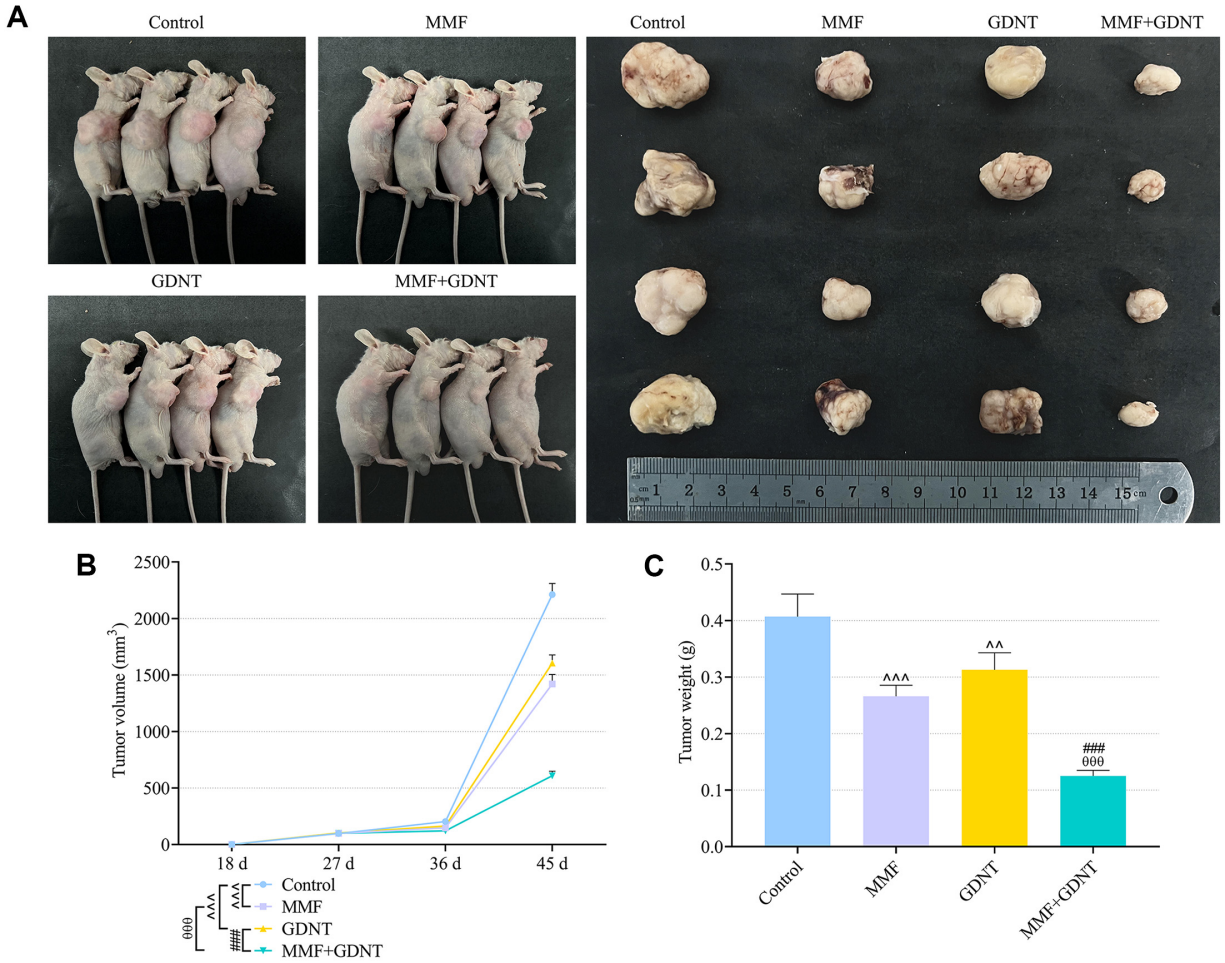
GDNT enhanced the effect of MMF on suppressing tumor growth of lung cancer in vivo. (**A**) Xenograft experiment was performed by subcutaneously injecting H1299 cells into mice, followed by treatment of MMF (20 mg/kg/2d) and/or GDNT (3 mg/kg/1d). After 45 days, all tumor-bearing mice were sacrificed, and the tumors were excised and photographed. (**B**) The volume of tumors was calculated. (**C**) The weight of tumors was measured. Data from all experiments performed thrice were expressed as mean ± standard deviation. ^^*p* < 0.01, ^^^*p* < 0.001, vs. Control; ^θθθ^*p* < 0.001, vs. MMF; ^###^*p* < 0.001, vs. GDNT.

**Fig. 8:**
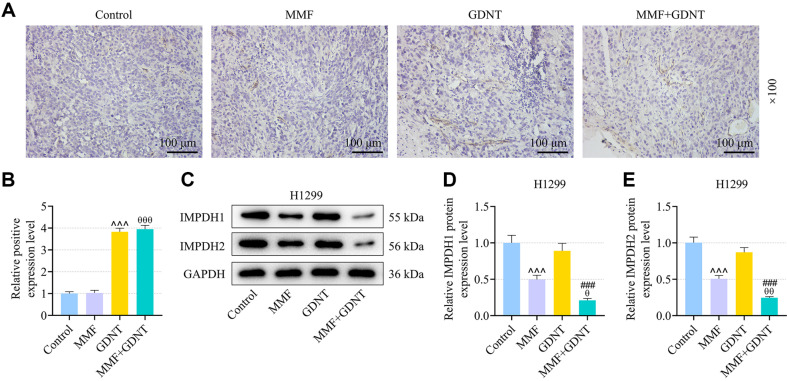
GDNT strengthened the effect of MMF on down-regulating CES2 and IMPDH1/2 expressions in lung cancer in vivo. (**A, B**) After xenograft experiment, immunohistochemistry was carried out to detect the expression of CES2 in the excised tumors. (**C-E**) Protein levels of IMPDH1 and IMPDH2 in the excised tumors were determined by Western blot. Relative expression levels were normalized with GAPDH. Data from all experiments performed thrice were expressed as mean ± standard deviation. ^^^*p* < 0.001, vs. Control; ^θ^*p* < 0.05, ^θθ^*p* < 0.01, vs. MMF; ^###^*p* < 0.001, vs. GDNT.

Considering the prominent role of GDNT in suppressing the development of lung cancer cells by increasing apoptosis, the regulatory effect of GDNT cooperated with MMF on tumorigenesis in lung cancer should be explored. Thus, we performed xenograft experiment through subcutaneously injecting H1299 cells into nude mice, followed by administration with GDNT and/or MMF. 45 days later, it was observed that either GDNT or MMF markedly reduced the volume and weight of tumors excised from the mice (Fig. 7A-7C, *p* < 0.01), and this reduction was more pronounced by co-administration with GDNT and MMF (Fig. 7A-7C, *p* < 0.001). As illustrated in Fig. 8A, the results of IHC identified that MMF administration exerted no significant effect on CES2 expression in mouse tumor tissues, but GDNT increased the expression of CES2 (Fig. 8A-8B, *p*<0.001). In addition, protein levels of IMPDH1 and IMPDH2 in mouse tumor tissues were markedly down-regulated by MMF (Fig. 8C-8E, *p* < 0.001), but were only fine-tuned by GDNT. Notably, this suppressive effect of MMF was potentiated by GDNT (Fig. 8C-8E, *p* < 0.01).

## Discussion

Currently, radiotherapy and first-line antineoplastic agents remain the basic treatments for most patients with advanced NSCLC, but the overall survival rate is affected by factors including side effects, non-targeted cytotoxicity and drug resistance [33]. With the rise of integrative therapies for malignant tumors, *G. lucidum* and its extracts have attracted increasing attention in anti-tumor and enhancing sensitivity to chemoradiotherapy, owing to their advantages of suitability for long-term use, low toxicity and multi-targets [13, 34]. The present study demonstrated that GDNT could induce cell cycle arrest to increase apoptosis of LUAD cells in vitro, and restrict tumor growth in LUAD tumor-bearing mice. Furthermore, the anti-cancer effect of MMF on LUAD was found to be potentiated in combination with GDNT, which was mechanically mediated by CES2.

Ample evidence at home and abroad has supported that *Ganoderma* triterpenoid plays a tumor-suppressive role in a variety of cancers by inducing cell apoptosis and suppressing cell proliferation, which are closely associated with the regulation of cell cycle and apoptosis-related signal pathways [35-37]. In the early study of breast cancer, Jiang *et al*. have identified that GDNT can reduce proliferation, migration and invasion of cancer cells by inhibiting cell division cycle 20 (CDC20) which is a key protein in manipulating the spindle mitotic checkpoint [15, 38]. The deficiency of CDC20 has been demonstrated to decrease the percentage of G0/G1 phase and increase the percentage of G2/M phase in cutaneous squamous cell carcinoma cells [39]. Targeting CDC20 is increasingly considered as a potential therapeutic strategy in several cancers including lung cancer [40]. In this study, we found that GDNT treatment (6.25 μM) evidently facilitated apoptosis and induced cell cycle arrest by decreasing the percentage of G0/G1 phase and increasing both percentages of S and G2/M phases in either H1299 or A549 cells, indicating that GDNT can be used as a novel drug against LUAD.

As an immunosuppressant, MMF, which can be hydrolyzed into MPA in the body, is widely used in the clinical treatment after solid organ transplantation to prevent rejection reaction through inhibiting IMPDH [41], but has been recently evidenced to repress the tumor progression via targeting tumor-associated fibroblast [31]. Although the anti-proliferative role of MMF has been confirmed in lung cancer, its underlying mechanism in LUAD is not fully expounded. Of note, this study unveiled that MMF treatment alone boosted apoptosis of LUAD cells and blocked intercellular DNA replication by augmenting the percentage of S phase, consistent with the findings of Ling *et al*. [42]. Importantly, the anti-cancer effect of MMF on LUAD cells was subsequently enhanced in the presence of GDNT. The above information indicated that GDNT and MMF can synergistically inhibit the growth of LUAD cells through inducing apoptosis and cell cycle arrest.

Based on the above-mentioned findings, we subsequently investigated the possible molecular mechanism of GDNT enhancing the anti-cancer effect of MMF. Through bioinformatics analysis, we found that CES2 was predicted as a potential target for GDNT. Reportedly, CES2 impacts the clinical results of cancer patients by affecting chemosensitivity [43]. In the study of graft rejection, CES2 has been suggested to function as a mediator in the metabolism of MMF to MPA [22]. In lung cancer cells, the expression of CES2 was increased by GDNT with/without MMF but was not significantly affected in the presence of MMF alone. MPA can target IMPDH which has been confirmed to be highly expressed in many tumors [44, 45]. In this study, the results of Western blot revealed that both IMPDH1 and IMPDH2 expressions were down-regulated by MMF in the cells, which was further strengthened by GDNT. IMPDH1 and IMPDH2 are two isoforms of IMPDH that is implicated in cell metabolism and proliferation by modulating *de novo* GTP biosynthesis [46]. Intriguingly, the increased expression of IMPDH is frequently confirmed in different cancers and correlated with tumorigenesis [47]. The analysis of UALCAN database in this study also confirmed high expressions of IMPDH1 and IMPDH2 in LUAD samples. Huang *et al*. have suggested that IMPDH inhibition combined with chemotherapy can improve survival of small cell lung cancer mouse models [48]. Additionally, targeting IMPDH to mediate cytoophidia assembly possibly improves anti-cancer treatment, but the effect of IMPDH on LUAD remains unclear [49]. In view of this, we surmised that GDNT may target CES2 to enhance the activation of MMF-MPA-IMPDH axis and suppress the progression of LUAD. Hence, we knocked down CES2 in H1299 or A549 cells to validate our speculation. As expected, the results of rescue assays revealed that CES2 knockdown reversed the synergistic effect of GDNT and MMF against lung cancer in vitro. After xenograft experiment, we further confirmed that GDNT can restrict tumor growth in mice injected with H1299 cells by activating CES2 to enhance metabolism of MMF into MPA.

## Conclusion

Despite the importance of IMPDH in anti-cancer treatment, the development and application of IMPDH inhibitors are limited by their side effects at high doses [25]. The present study unravels the anti-cancer mechanism of GDNT in LUAD, and further provides a new direction for developing adjuvant agents to enhance the efficacy of IMPDH inhibitors in treating lung cancer.

## Supplemental Materials

Supplementary data for this paper are available on-line only at http://jmb.or.kr.
