# Supplementary material for: Ganodermanontriol Suppresses the Progression of Lung Adenocarcinoma by Activating CES2 to Enhance the Metabolism of Mycophenolate Mofetil
Source: J Microbiol Biotechnol. 2023 Oct 1;34(2):249–61. doi: 10.4014/jmb.2306.06020 (PMC10940751; doi:10.4014/jmb.2306.06020)
Supplement: Supplementary file 1 [file jmb-34-2-249-supple.pdf]

J. Microbiol. Biotechnol.
https://doi.org/10.4014/jmb.2306.06020

Target	Common name	Uniprot ID	ChEMBL ID	Target Class	Probability*	Known actives (3D/2D)
Carboxylesterase 2	CES2	O00748	CHEMBL3180	Enzyme	0.244884958181	8 / 14
TNF-alpha	TNF	P01375	CHEMBL1825	Secreted protein	0.161765714709	3 / 6
DNA topoisomerase II alpha	TOP2A	P11388	CHEMBL1806	Isomerase	0.145150143153	3 / 2
Glucocorticoid receptor	NR3C1	P04150	CHEMBL2034	Nuclear receptor	0.128531577649	86 / 38
Androgen Receptor	AR	P10275	CHEMBL1871	Nuclear receptor	0.128531577649	139 / 127
Protein farnesyltransferase	FNTA FNTB	P49354 P49356	CHEMBL2094108	Enzyme	0.128531577649	28 / 9
Cytochrome P450 19A1	CYP19A1	P11511	CHEMBL1978	Cytochrome P450	0.120225750913	20 / 272
Protein-tyrosine phosphatase 1B	PTPN1	P18031	CHEMBL335	Phosphatase	0.120225750913	108 / 57
Estrogen receptor beta	ESR2	Q92731	CHEMBL242	Nuclear receptor	0.120225750913	34 / 44
Estrogen receptor alpha	ESR1	P03372	CHEMBL206	Nuclear receptor	0.120225750913	31 / 38
Niemann-Pick C1-like protein 1	NPC1L1	Q9UHC9	CHEMBL2027	Other membrane protein	0.120225750913	16 / 11
Prostaglandin E synthase	PTGES	O14684	CHEMBL5658	Enzyme	0.120225750913	13 / 13
HMG-CoA reductase	HMGCR	P04035	CHEMBL402	Oxidoreductase	0.120225750913	203 / 76
Cytochrome P450 17A1	CYP17A1	P05093	CHEMBL3522	Cytochrome P450	0.120225750913	58 / 42
